# Butyrate Prevents Obesity Accompanied by HDAC9-Mediated Browning of White Adipose Tissue

**DOI:** 10.3390/biomedicines13020260

**Published:** 2025-01-21

**Authors:** Jing Yang, Guoli Li, Shan Wang, Mingqian He, Sijing Dong, Ting Wang, Binyin Shi, Patrick C. N. Rensen, Yanan Wang

**Affiliations:** 1 Department of Endocrinology, First Affiliated Hospital of Xi’an Jiaotong University, Xi’an 710061, China; doris_yangjing@163.com (J.Y.);; 2 Med-X Institute, Center for Immunological and Metabolic Diseases, First Affiliated Hospital of Xi’an Jiaotong University, Xi’an 710061, China; 3 Department of Cardiovascular Medicine, Shaanxi Provincial People’s Hospital, Xi’an 710061, China; 4 Department of Medicine, Division of Endocrinology, and Einthoven Laboratory for Experimental Vascular Medicine, Leiden University Medical Center, P.O. Box 9600, 2300 Leiden, The Netherlands

**Keywords:** obesity, butyrate, thermogenesis, HDAC9

## Abstract

Background/Objectives: Mounting evidence indicates that the short-chain fatty acid butyrate protects against obesity and associated comorbidities, partially through the induction of adipose tissue thermogenesis. However, the effects of butyrate on white adipose tissue (WAT) browning and its molecular mechanism are still elusive. The objective of this study was to investigate butyrate-induced thermogenesis in white adipose tissue and its underlying mechanism. Methods: We studied the effects of butyrate on diet-induced obesity in the humanized APOE*3-Leiden.CETP transgenic mouse model and explored factors related to white adipose browning. Specifically, mice were challenged with a high-fat diet supplemented with butyrate. Adiposity was measured to assess obesity development. Energy metabolism was detected using an indirect calorimetry system. RNA-seq analysis was conducted to analyze the transcription landscape of WAT and responsible targets. Furthermore, the revealed molecular mechanism was verified in vitro. Results: Butyrate alleviated high-fat diet-induced obesity and promoted energy expenditure accompanied by brown adipose tissue activation and WAT browning. Mechanistically, RNA-seq analysis revealed that butyrate downregulated HDAC9 in WAT. Additionally, butyrate decreased HDAC9 while increasing thermogenesis in vitro. Inhibition of HDAC9 with TMP269 promoted thermogenic gene expression, mimicking the effects of butyrate. Conclusions: Butyrate protects against diet-induced obesity accompanied by decreasing the expression of HDAC9 in white adipose tissue and inducing browning. This study reveals a new mechanism whereby butyrate activates adaptive thermogenesis and provides new insights for the development of weight-loss drugs targeting adipose HDAC9.

## 1. Introduction

Obesity has been an epidemic worldwide for decades, becoming an intractable healthcare issue associated with a wide spectrum of metabolic disturbances such as type 2 diabetes, atherosclerotic cardiovascular disease, and metabolic dysfunction-associated steatotic liver disease (MASLD) [1]. The hallmark of obesity pathology is an imbalance between energy intake and energy expenditure. Hence, to combat obesity, numerous efforts have been made to restore energy homeostasis through lifestyle interventions, bariatric surgery, and anti-obesity medications. However, the efficacy of current anti-obesity management is unstable across populations, and more therapeutic strategies are still required to overcome the escalating prevalence.

Adipose tissue plays a pivotal role in energy metabolism and obesity development. In mammals, adipose tissue is typically divided into white adipose tissue (WAT) and brown adipose tissue (BAT), which primarily store surplus energy in the form of triglyceride and dissipate energy as heat, respectively. In addition, “brown-like” adipocytes, termed “beige/brite” adipocytes, can be induced in WAT by stimuli such as cold exposure and catecholamines. This process is regarded as WAT browning. Brown and beige adipocytes can produce heat via uncoupling protein 1 (UCP1) through adaptive thermogenesis. Accumulating evidence has shown that promoting adipose tissue thermogenesis via BAT activation [2] and WAT browning [3] are promising strategies to treat obesity. Since the mass and activity of BAT are limited in adults, WAT browning seems to be a more promising strategy to fight obesity and related metabolic diseases due to its considerable amount and flexibility in humans with obesity [4].

The short-chain fatty acid butyrate is a metabolite of intestinal flora mainly produced by the fermentation of dietary fiber in the colon. Emerging studies have shown that butyrate has beneficial effects on energy homeostasis and body weight control. By oral, intraperitoneal, or rectal administration, butyrate can effectively prevent and treat obesity and related disorders in different animal models [5,6]. In humans, cross-sectional studies indicate that plasma butyrate is inversely associated with body mass index (BMI) [7]. The abundance of butyrate-producing taxa and the expression of butyrate-production genes in the gut microbiome are reduced in individuals with obesity. Some clinical trials also indicate that butyrate is efficient in combating obesity in children [8]. Mechanistically, butyrate is capable of reducing energy intake by suppressing appetite, as well as promoting energy expenditure via adipose tissue thermogenesis [9]. However, the exact molecular mechanism of how butyrate modulates adaptive thermogenesis, in particular via regulation of browning of WAT to dissipate energy, is still largely unknown.

Histone deacetylases (HDACs), which are epigenetic modifying enzymes catalyzing the deacetylation of proteins, have emerged as pivotal epigenetic regulators involved in energy homeostasis. A total of 18 HDACs have been described in mammals, categorized into four classes, namely Class I (HDAC1, 2, 3, 8), Class IIa (HDAC4, 5, 7, 9), Class IIb (HDAC6, 10), Class III (NAD+ dependent Sirtuin deacetylase, SIRT1-7), and Class IV (HDAC11) [10]. Recent studies demonstrate that HDACs can function as adaptive thermogenesis molecular brakes via modulating gene transcription in both a deacetylation-dependent or -independent manner [11]. For instance, HDAC1 [12], HDAC11 [13], and HDAC6 [14] exert inhibitory effects on adaptive thermogenesis, while HDAC3 [15] and SIRT6 [16] are required to maintain the thermogenic function of BAT. Butyrate is commonly considered an inhibitor of Class I and II HDACs [17]. Through inhibiting HDAC activity, butyrate is involved in gut homeostasis [17], contributes to colorectal cancer management [18], benefits rheumatoid arthritis [19], mitigates non-alcoholic steatohepatitis [20], protects pancreatic beta cell dysfunction [21], improves post-myocardial infarct tissue repair [22], as well as reinstates learning ability in neurodegeneration [23]. However, whether the HDAC inhibitory action of butyrate may also be responsible for adipose tissue thermogenesis remains elusive.

In this research, we studied the anti-obesity effects of butyrate in the high-fat diet-induced obese humanized APOE*3-Leiden.CETP mouse model. Using RNA-seq analysis, we explored the gene transcription landscape of both WAT and BAT, revealing that butyrate inhibited HDAC9 in WAT. Furthermore, we verified the stimulating effects of HDAC9 inhibition on adipocyte thermogenesis in vitro. These results provide new insights into the molecular mechanism of how butyrate is involved in WAT browning.

## 2. Materials and Methods

### 2.1. Animal Study

Hemizygous APOE*3-Leiden (E3L) mice were crossbred with homozygous human cholesteryl ester transfer protein (CETP) transgenic mice to generate heterozygous E3L.CETP mice as described in [24]. Mice were group-housed in individually ventilated cages at a 22 °C room temperature with 40% relative humidity under a 12 h/12 h light/dark cycle, with access to standard rodent chow diet and water ad libitum, unless indicated. After 1 week of acclimatization, at the age of 10–14 weeks, male E3L.CETP mice were randomly divided into two groups and fed either a high-fat diet (HFD) containing 60% calories from fat without (vehicle group) or with 5% (*w*/*w*) sodium butyrate (Sigma Aldrich; butyrate group) for 9 weeks. At the end of the experiment, mice were euthanized with 10% chloral hydrate (400 mg/kg, i.p), perfused with ice-cold PBS via cardiac perfusion for 5 min, and various organs were collected for further analysis. The animal study was conducted in compliance with the National Institutes of Health guidelines and was approved by the Animal Care and Use Committee of Xi’an Jiaotong University.

Body weight and body composition: The body weight of all mice was measured weekly throughout the study. Body composition analysis was performed before and after 5 and 9 weeks of exposure to the HFD using a Nuclear Echo Magnetic Resonance Imaging System (EchoMRI-100; EchoMedical Systems, USA). All measurements were taken at 10:00 AM of the day.

Indirect calorimetry: Indirect calorimetry was carried out in calorimetric chambers controlled for light, temperature, and humidity (TSE Systems GmbH, Germany). Mice fed an HFD for 1 week with or without butyrate supplementation were acclimated in the chambers for 3 days before recording. O2 consumption (mL/h) and CO2 production (mL/h) were recorded, from which the respiratory exchange rate (RER) and energy expenditure were calculated. Meanwhile, locomotor activity was assessed. The temperature in the calorimetric chambers was first fixed at 22 °C for 48 h during a 12 h/12 h light/dark cycle. When recording under cold exposure, the temperature was decreased to 4 °C for another 24 h. Indirect calorimetry was repeated before the mice were sacrificed.

Serum biochemistry: Mice were fasted for 5 h with food withdrawal at 9:00 AM, and total blood was collected from the tail vein with EDTA-coated tubes. Blood samples were then centrifuged at 5000 rpm for 20 min at 4 °C, and plasma was isolated for analysis. Plasma triglycerides, cholesterol, and glucose were measured using standard enzymatic kits from Roche Diagnostics (Triglycerides: #20767107322, Cholesterol Gen.2: #03039773190, and Glucose HK Gen.3: #04404483190; Mannheim, Germany) according to the manufacturer’s instructions.

Histological measurement of tissue: Adipose tissue was fixed with 4% paraformaldehyde fix solution for 48 h, gradually dehydrated in ethanol, and embedded in paraffin blocks. For H&E staining, paraffin sections (5 μm) were first incubated with hematoxylin for 5 min to stain nuclei. After washing with water, sections were immersed in eosin for 15 s, re-washed, dried at 55 °C, and fixed with neutral resin sealing tablets. Staining for UCP1 (1:4000; Ab10983, Abcam, Waltham, MA, USA) was conducted as described previously [8]. In the end, sections were photographed with a light microscope.

### 2.2. Cell Culture and Treatment

3T3-L1 pre-adipocytes: 3T3-L1 pre-adipocytes were cultured in growth medium (GM; high-glucose DMEM supplemented with 10% fetal bovine serum (FBS), 1% penicillin, and streptomycin) in a humidified atmosphere of 5% CO_2_ at 37 °C. Two days post-confluence, cells were differentiated into adipocytes using differentiation medium (DM; DMEM containing 10% FBS, 1% penicillin and streptomycin, 1 μM dexamethasone (Sigma-Aldrich, Inc., St Louis, MO, USA), 0.5 mM 3-isobutyl-1-methylxanthine (IBMX, Sigma-Aldrich, Inc., St Louis, MO, USA), 5 μM rosiglitazone (Sigma-Aldrich, Inc., St Louis, MO, USA), and 5 μg/mL human insulin (Solarbio)) along with phosphate-buffered saline (PBS; as control), butyrate (Sigma-Aldrich, Inc., St Louis, MO, USA), HDAC9 inhibitor (TMP269, MedChemExpress, Monmouth Junction, NJ, USA), or their combination in DM for 48 h. To explore the effect of butyrate on mature adipocytes, 3T3-L1 pre-adipocytes were differentiated for 6 days and then treated with PBS, butyrate, or TMP269 for 24 h. All mentioned concentrations of reagents refer to their working concentrations.

Adipose stromal vascular fraction (SVF) isolation and differentiation: Subcutaneous white adipose tissue (sWAT) was dissected from 5-week-old C57/BL6J male mice, minced, and digested in Liberase (Sigma-Aldrich, USA) at 37 °C for 45 min with gentle agitation. After passing through a cell strainer, the SVF was pelleted by centrifuging at 400× *g* for 5 min at 4 °C. The pellet was then resuspended and plated in growth medium. For differentiation, post-confluent primary preadipocytes were induced with differentiation medium supplemented with indicated compounds as shown above. For mature adipocytes, primary preadipocytes were differentiated for 10 days and then treated with PBS, butyrate, or TMP269 for 24 h.

### 2.3. Total RNA Extraction and RT-qPCR

The total mRNA of collected adipose tissue and cultured cells was extracted with TRIzol reagent (Thermo Scientific, Waltham, MA, USA). Then, 1 μg of total RNA was used to synthesize cDNA using the High-Capacity cDNA Reverse Transcription Kit (Thermo Scientific, USA) according to the manufacturer’s instructions. Gene expression was evaluated by quantitative real-time PCR (RT-qPCR) using the SYBR Green qPCR Master Mix (Thermo Scientific, USA). With Cyclophilin A as the reference gene, the relative value was calculated using the ΔΔCT method. For primer sequences, refer to Supplementary Table S1.

### 2.4. RNA Sequencing Analysis

Total RNA was first assessed for quantity and quality using the RNA Nano 6000 Assay Kit of the Bioanalyzer 2100 system (Agilent Technologies, Santa Clara, CA, USA). Then mRNA was purified from total RNA using poly-T oligo-attached magnetic beads. cDNA synthesis was subsequently performed and the library fragments were purified with AMPure XP system (Beckman Coulter, Beverly, MA, USA) to select cDNA fragments of preferentially 370~420 bp in length. Afterward, the clustering of the index-coded samples was performed on a cBot Cluster Generation System using TruSeq PE Cluster Kit v3-cBot-HS (Illumia) according to the manufacturer’s instructions. After cluster generation, the library preparations were sequenced on an Illumina NovaSeq platform, and 150 bp paired-end reads were generated. Raw data (raw reads) in FASTQ format were firstly processed through fastp software to get clean reads. Paired-end clean reads were aligned to the reference genome against mouse reference (mm39/mGRC39) using Hisat2 v2.0.5. The mapped reads of each sample were assembled by StringTie (v1.3.3b) in a reference-based approach. FeatureCounts v1.5.0-p3 was used to count the reads numbers mapped to each gene. Then, the FPKM of each gene was calculated. Differential expression analysis was performed using the DESeq2 R package (1.20.0). The *p*-values were adjusted using the Benjamini–Hochberg method. A corrected *p*-value of 0.05 and an absolute fold change of 2 were set as the threshold for significantly differential expression. Gene Ontology (GO) enrichment analysis of differentially expressed genes was implemented by the clusterProfiler R package. GO terms with a corrected *p*-value less than 0.05 were considered significantly enriched by differentially expressed genes. The statistical enrichment of differentially expressed genes in the KEGG pathway was analyzed using clusterProfiler R package.

### 2.5. Western Blotting Analysis

Adipose tissue and cultured adipocytes were collected and homogenized in RIPA lysis buffer with phosphatase and protease inhibitors to obtain protein extracts. After centrifugation at 12,000 rpm for 30 min at 4 °C, the supernatants were isolated and protein concentrations were detected using the BCA Protein Assay Kit (Thermo Scientific, USA). A total of 20 μg of protein per sample was separated by 10% SDS-PAGE gel and transferred onto a polyvinylidene difluoride (PVDF) membrane (Millipore, Billerica, MA, USA). After sealing with 5% skim milk powder for 1 h, the membrane was incubated overnight at 4 °C with primary antibody: uncoupling protein-1 (UCP1, 1:1000, #10983, Abcam) and β-actin (1:3000, #4967, Cell Signaling Technology, Danvers, MA, USA). Afterward, membranes were washed with Tris-buffered saline with 0.01% Tween (TBST) and then incubated with the secondary antibody (goat anti-rabbit, 1:5000, Cell Signaling Technology, Danvers, MA, USA) at room temperature for 1 h. After washing with TBST three times, the bands were visualized by chemiluminescence (ECL, Thermo Scientific) and exposed on an Imaging System (Biorad). Protein Bands were quantified using ImageJ software, and β-actin protein was used as an internal control.

### 2.6. Statistical Analysis

Statistical analysis was performed using GraphPad Prism 9 software (San Diego, CA, USA). All data were presented as mean ± SEM. Statistical tests for each comparison are shown in the legends corresponding to the specific figure. Briefly, differences between the two groups were determined using the unpaired two-tailed Student’s *t*-test. Differences among three or more groups were determined by one-way ANOVA analysis of variance followed by Tukey’s post hoc test. When considering intervention (with or without butyrate) and time of day (light or dark) as factors, two-way ANOVA was carried out to analyze indirect calorimetry. In all cases, *p* < 0.05 was considered statistically significant.

## 3. Results

### 3.1. Butyrate Supplementation Alleviates High-Fat Diet-Induced Obesity in Humanized APOE*3-Leiden.CETP Double Transgenic Mice

**Figure 1:**
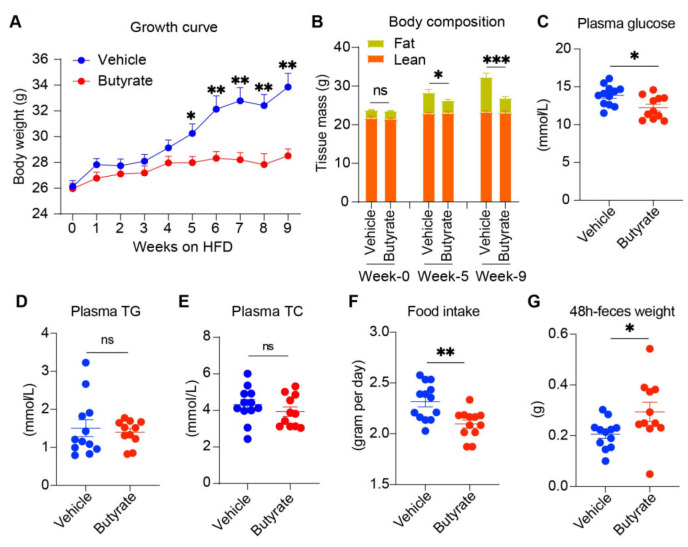
Butyrate supplementation alleviates high-fat diet-induced obesity. Fourteen- to sixteen-week-old APOE*3-Leiden.CETP mice were fed a 60% high-fat diet without or with 5% (*w*/*w*) sodium butyrate (defined as the vehicle and butyrate groups, respectively) for 9 weeks. Body weight was measured weekly (**A**). Body composition, including fat mass and lean mass, was measured by EchoMRI at indicated time points (**B**). Plasma glucose (**C**), triglyceride (TG) (**D**), and total cholesterol (**E**) were assessed at the end of the intervention. Food intake (**F**) and the amount of feces during 48 h (**G**) were weighted. Data are shown as means ± SEM (n = 11–12). Unpaired two-tailed Student’s *t*-test was used. * *p* < 0.05, ** *p* < 0.01, *** *p* < 0.001, ns = not significant.

We first assessed the effects of butyrate on obesity development. During the 9-week intervention period, butyrate supplementation largely prevented HFD-induced obesity, as evidenced by attenuated body weight gain (Figure 1A) and fat mass gain (Figure 1B), without affecting lean mass (Figure 1B). Concurrently, butyrate decreased plasma glucose levels (Figure 1C), while plasma triglyceride (Figure 1D) and total cholesterol levels (Figure 1E) were comparable between the two groups. In addition, we observed that butyrate decreased food intake (Figure 1F), which was in line with our previous study [9], and increased fecal excretion (Figure 1G). Collectively, these data demonstrated that butyrate administration prevented diet-induced body weight gain and hyperglycemia. These effects were partly ascribed to suppressed appetite as well as energy intake, as evidenced by increased defecation.

### 3.2. Butyrate Consumption Promotes Energy Expenditure and Lipid Oxidation to Resist Obesity

**Figure 2:**
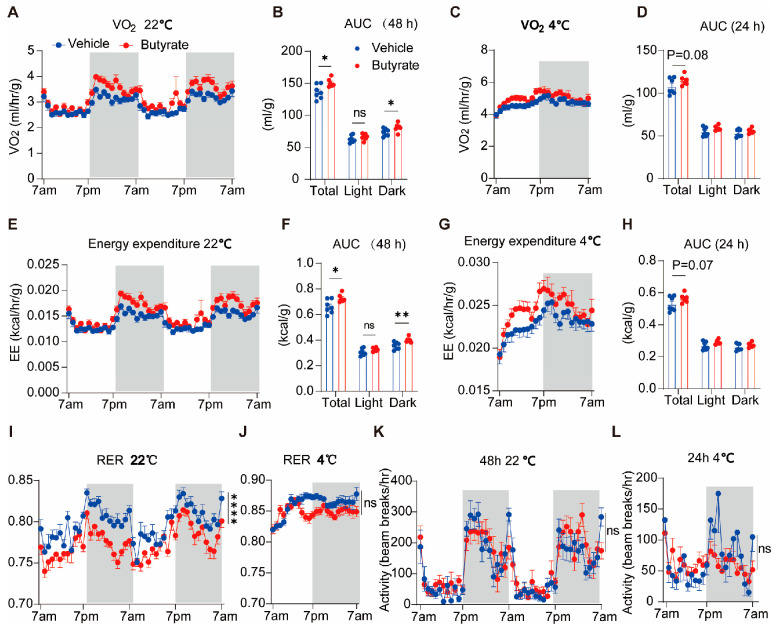
Butyrate supplementation increases energy expenditure at both room temperature and under cold exposure. After 1 week of HFD intervention with or without butyrate, mice were housed in a PromethION metabolic measurement system at 22 °C and 4 °C, respectively. After 3 days of acclimatization, the oxygen consumption (**A**–**D**), energy expenditure (**E**–**H**), respiratory exchange ratio (RER; **I**,**J**), and physical activity (**K**,**L**) were monitored and calculated. Data are shown as means ± SEM (n = 6–7) during a 48 h cycle at 22 °C and a 24 h cycle at 4 °C. Two-way ANOVA was used. * *p* < 0.05, ** *p* < 0.01, ns = not significant.

To further analyze the effects of butyrate on energy metabolism, we performed indirect calorimetry during the first week of the intervention when the body weight difference was still negligible. Mice were housed in a fully automated PromethION metabolic measurement system and energy metabolism was assessed after acclimatization at both regular room temperature (22 °C) and under cold exposure (4 °C). At 22 °C, butyrate increased O_2_ consumption (Figure 2A,B) and energy expenditure (Figure 2E,F), specifically in the active dark period. Unexpectedly, these effects were not amplified at 4 °C (Figure 2C,D,G,H). In addition, compared with the control group, butyrate lowered the respiratory exchange ratio (RER) at both 22 °C (Figure 2I) and 4 °C (Figure 2J), implying increased fat oxidation at the expense of carbohydrate oxidation. However, butyrate did not affect physical activity at either ambient temperature (Figure 2K,L). The results were verified again before the mice were sacrificed (Figure S1). These data indicated that butyrate promoted energy expenditure and lipid oxidation by shifting the utilization of nutrients to combat obesity.

### 3.3. Butyrate Activates Thermogenesis in Brown Adipose Tissue and Induces Browning of White Adipose Tissue

**Figure 3:**
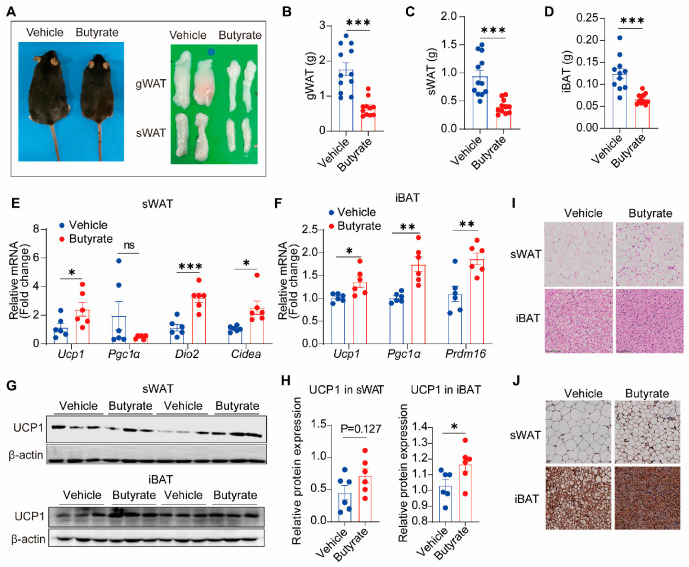
Butyrate supplementation increases markers of thermogenesis in brown and white adipose tissue. After 9 weeks of intervention, WAT depots were excised, and representative images of mice and gonadal white adipose tissue (gWAT) and subcutaneous white adipose tissue (sWAT) are shown (**A**). Weights of gWAT (**B**), sWAT (**C**), and interscapular brown adipose tissue (iBAT) (**D**) were measured. mRNA expression of thermogenic markers was analyzed using qPCR in sWAT (**E**) and iBAT (**F**). UCP1 protein levels were assessed by western immunoblotting analysis of sWAT (**G**) and iBAT (**H**). Representative stainings for H&E (**I**) and UCP1 (**J**) of sWAT and iBAT are also depicted. Data are shown as means ± SEM (n = 6, 11–12). Unpaired two-tailed Student’s *t*-test was used. * *p* < 0.05, ** *p* < 0.01, *** *p* < 0.001, ns = not significant.

In accordance with decreased food intake and increased lipid oxidation, butyrate caused leaner body weight with smaller fat depots (Figure 3A). Consistently, butyrate obviously reduced the mass of gWAT, sWAT, and interscapular brown adipose tissue (iBAT) (Figure 3B–D), implying reduced adiposity. Since thermogenesis plays a pivotal role in adipose tissue metabolism and energy homeostasis, we measured thermogenic markers in sWAT and iBAT. Butyrate upregulated expression of Ucp1, deiodinase iodothyronine type 2 (Dio2), cell death-inducing DNA fragmentation factor alpha subunit-like effector A (Cidea) in sWAT (Figure 3E) and Ucp1, peroxisome proliferator-activated receptor gamma coactivator 1 alpha (Pgc1a), and PR domain containing 16 (Prdm16) in iBAT (Figure 3F). Also, butyrate increased protein levels of UCP1 in iBAT and showed a rising trend in sWAT, as assessed by western blotting analysis (Figure 3G,H). Accordingly, histological analysis with H&E staining revealed that butyrate reduced adipocyte size in sWAT and iBAT (Figure 3I). Immunohistochemical analysis of UCP1 staining indicated that butyrate increased the UCP1 expression in sWAT and iBAT (Figure 3J). Collectively, these data suggest that butyrate activates thermogenesis in BAT and induces browning of WAT, both of which likely contribute to the anti-obesity effects of butyrate. In addition, we also assessed the effects of butyrate on adipogenesis, adipokine production, lipogenesis, lipolysis, glucose uptake, fatty acid transport, and inflammation of sWAT and found that butyrate upregulated the genes involved in adipokine production and glucose metabolism (Figure S2).

### 3.4. Butyrate Remodels Global Transcriptome Landscape of Adipose Tissue and Reduces HDAC9 Expression in sWAT

**Figure 4:**
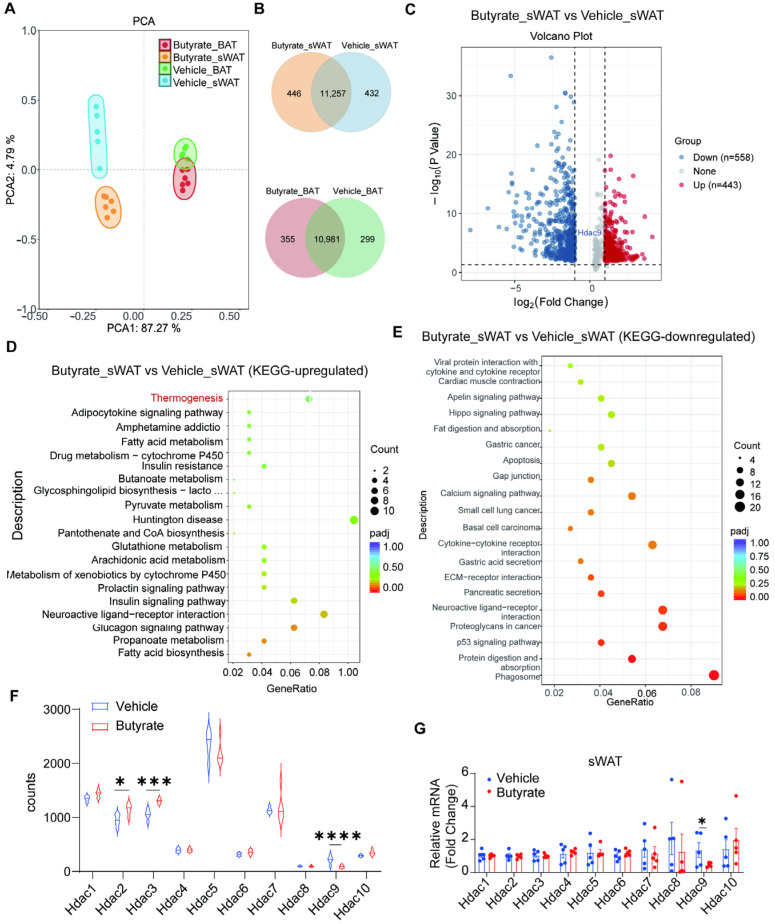
Butyrate supplementation markedly remodels the global transcriptome landscape of iBAT and sWAT. Mice were fed a high-fat diet and treated with or without butyrate for 9 weeks, after which principal component analysis on expression of genes with average FPKM (Fragments Per Kilobase Million) ≥1 was performed in BAT and sWAT (**A**). Venn diagrams comparing overlap and differences between butyrate intervention and the vehicle in sWAT and BAT (**B**). Heat map depicting the profile of differential gene expression in sWAT (**C**). Enriched KEGG terms of sWAT that were significantly higher expressed in the butyrate group (**D**). Enriched KEGG terms of sWAT that were significantly lower expressed in the butyrate group (**E**). The counts of Hdac1-10 by RNA sequencing (**F**). Relative mRNA of Hdacs in sWAT determined by RT-qPCR (**G**). Data are shown as means ± SEM (n = 5–6). Unpaired two-tailed Student’s *t*-test was used in (**F**). * *p* < 0.05, *** *p* < 0.001, **** *p* < 0.0001.

To explore the effects of butyrate on adipose tissue metabolism from an unbiased molecular perspective, we performed RNA sequencing analysis of iBAT and sWAT in mice treated with or without butyrate. Principal component analysis clearly separated iBAT and sWAT into distinct clusters. Butyrate intervention caused a clear shift in clusters of both iBAT and sWAT (Figure 4A) and clearly remodeled the gene transcriptome of both adipose tissue depots, as shown by Venn diagrams (Figure 4B). Differential gene expression analysis revealed that butyrate upregulated the expression of 443 genes and downregulated 558 genes, including HDAC9 (Log2 FC = −1.29, FDR = 1.25 × 10^−5^) in sWAT compared to the vehicle (Figure 4C). KEGG pathway analysis of modulated genes in sWAT demonstrated that butyrate augmented the thermogenesis pathway (Figure 4D) and down-regulated some other metabolic pathways (Figure 4E). Also, butyrate exerted diverse functions on gene transcription of BAT (Figure S3). Given that butyrate has been recognized as an HDAC inhibitor targeting Class I and II HDACs, we re-evaluated the expression levels of these HDAC members from the obtained datasets (Figure 4F) and further validated the results with qPCR (Figure 4G) in sWAT. While the RNA sequencing data revealed upregulated HDAC2 and HDAC3 (Figure 4G), butyrate appeared to selectively decrease the mRNA expression of HDAC9 (Figure 4F,G), indicating that HDAC9 inhibition may be a plausible cellular mechanism underlying the regulation of butyrate on WAT browning.

### 3.5. HDAC9 Inhibitor TMP269 Mimics Butyrate to Promote Adipocyte Thermogenesis and Adipogenesis In Vitro

**Figure 5:**
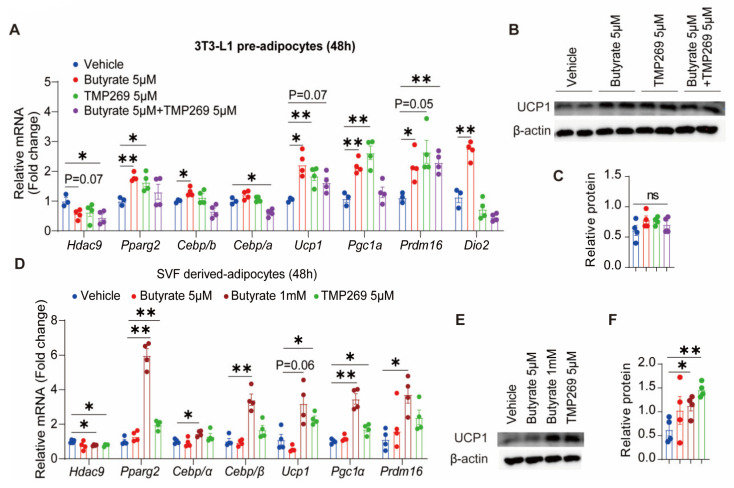
Butyrate and the HDAC9 inhibitor TMP269 steer pre-adipocytes toward a thermogenic phenotype and increase adipogenesis in vitro. 3T3-L1 pre-adipocytes were treated with 5 µM butyrate, 5 μM HDAC9 inhibitor TMP269, or both, for 48 h during cell differentiation. mRNA levels were analyzed by qPCR (**A**), and UCP1 protein levels were assessed with western blotting analysis (**B**) and quantified with ImageJ (**C**). Stromal vascular fraction (SVF) of mouse sWAT was isolated and cultured to induce adipocytes and treated with butyrate (5 µM and 1 mM) and TMP269 (5 µM) during differentiation. After 48 h of treatment, gene expression was tested by qPCR (**D**), and UCP1 protein levels were assessed with western blotting analysis (**E**) and quantified with ImageJ (**F**). Data were shown as mean ± SEM (n = 3–4). One-way ANOVA was used. * *p* < 0.05, ** *p* < 0.01, ns = not significant.

To reveal the function of HDAC9 on adipocyte metabolism, we tested the effect of HDAC9 inhibitor TMP269 on two white adipocyte models in vitro, in the presence or absence of butyrate. In 3T3-L1 pre-adipocytes, butyrate at a physiological concentration of 5 µM inhibited HDAC9 gene expression while promoting mRNA expression of adipogenesis markers, including peroxisome proliferator-activated receptor gamma (Pparg2), CCAAT/enhancer binding protein beta (Cebp/b), CCAAT/enhancer binding protein alpha (Cebp/a), and thermogenic markers, including Ucp1, Pgc1a, Prdm16, and Dio2 (Figure 5A). The HDAC9 inhibitor TMP269 displayed similar effects on adipogenesis and thermogenesis as compared to butyrate, while the combined application of butyrate and TMP269 did not show enhanced efficacy (Figure 5A). Consistent with mRNA expression, butyrate and TMP269 alone tended to increase the protein expression of UCP1, while co-treatment did not amplify efficacy (Figure 5B,C). To further substantiate these findings and shed light on the effects of HDAC9 on thermogenesis and adipogenesis in primary adipocytes, we isolated subcutaneous white adipose tissue from mice and extracted SVF to generate primary adipocytes. During the first 48 h of adipocyte differentiation, butyrate, TMP269, and their combination were added to the medium. Similar to findings in 3T3-L1 pre-adipocytes, butyrate downregulated HDAC9 mRNA expression, and both butyrate and TMP269 upregulated mRNA expression of adipogenesis and thermogenesis markers in SVF-induced adipocytes (Figure 5D). Butyrate and TMP269 also enhanced the protein level of UCP1 (Figure 5E,F). Meanwhile, we assessed the effects of butyrate and TMP269 on mature 3T3-L1 adipocytes and SVF-derived adipocytes for 24 h. Consequently, butyrate had no effect on differentiated 3T3-L1 adipocytes nor SVF-induced adipocytes, while TMP269 increased adipogenic genes of 3T3-L1 adipocytes (Figure S4). These in vitro data indicate that butyrate inhibits HDAC9 while promoting thermogenesis at the early stage of adipocyte differentiation. Furthermore, the HDAC9 inhibitor TMP269 mimics butyrate to steer adipocytes into a thermogenic phenotype and increase adipogenesis.

## 4. Discussion

Butyrate is the most extensively studied SCFA, with clear beneficial effects on obesity and insulin sensitivity. In this study, we demonstrate consistent benefits of butyrate on adiposity in a high-fat diet-induced humanized APOE*3-Leiden.CETP mouse model. Moreover, we indicate that butyrate intervention prevented obesity accompanied by WAT browning, mechanistically associated with decreased HDAC9 in WAT. Notably, the inhibition of HDAC9 is validated to induce adipocyte thermogenesis in vitro.

Gut microbiota is well established to be involved in host adaptive thermogenesis [25]. Short-chain fatty acids (SCFAs), primarily encompassing acetate, propionate, and butyrate, facilitate the crosstalk between gut microbiota and the host, thus playing a pivotal role in whole-body metabolism [26]. Evidence indicates that SCFAs have the potential to mitigate obesity and its metabolic complications. For instance, it is well documented that oral administration of butyrate could significantly alleviate high-fat diet-induced obesity, although it does not fully restore the phenotype exactly to that observed in the mice fed a standard chow diet [27]. In terms of mechanism, animal studies indicate that butyrate affects both sides of the energy equation, including energy intake and energy expenditure. As shown in this study and our previous research [9], butyrate inhibits food intake. This is mediated by inducing satiety through the gut-brain neural circuit [9], gut hormones (i.e., GLP-1, GIP, and PYY) [28], as well as reward regulation of the opioidergic system [29]. However, inhibition of food intake is not the unique mechanism since we find butyrate still prevents diet-induced weight gain in pair-fed mice [9]. Meanwhile, our study indicates that mice treated with butyrate defecated more, implying reduced energy intake along with food inhibition (Figure 1G). Since butyrate is well established to maintain the colonic epithelium and enhance intestinal barrier function, increased defecation is not possible due to affected absorption capability or localized inflammation. We speculate that it may result from enhanced motility of the intestine, while more evidence is needed.

Here, we show that promoting energy burning, via activating adipose tissue thermogenesis in both BAT and sWAT, is another pivotal mechanism through which butyrate effectively mitigates obesity. Butyrate intervention significantly increased the energy expenditure at room temperature, while the effect was not amplified at 4 °C (Figure 2). We speculate that this is because cold stimulation largely activates the adaptive thermogenesis in both butyrate and control groups, shielding the effect of butyrate. In our previous study, we revealed butyrate activates BAT via the brain-BAT axis through increasing sympathetic outflow [9]. Another group reported that butyrate can be taken up by adipocytes to promote the transcription of the thermogenic marker UCP1 [30]. While most research on the thermogenic effects of butyrate has focused on BAT activation, the effects of butyrate on the browning of WAT and its underlying mechanism are largely unknown. In fact, WAT browning appears to be a more attractive strategy to treat obesity in clinical application, considering the restricted mass and functional inactivity of BAT in adults. In this study, we show that butyrate also induces the expression of thermogenic markers, including UCP1, in WAT, implying WAT browning.

As for the underlying mechanism, we demonstrate that HDAC9 is involved in the butyrate-intervened WAT browning. Given the fact that butyrate is an HDAC inhibitor [31] and HDACs play a pivotal role in thermogenesis, we investigated whether the effects of butyrate on WAT browning were associated with HDAC inhibition. To this end, we conducted RNA-seq analysis of sWAT and quite surprisingly found that butyrate only downregulated the expression of HDAC9 among all members of the HDAC family. Next, we explored the effects of butyrate on HDAC9 as well as the function of HDAC9 on adipocyte thermogenesis in different cell models. Besides reducing HDAC9 gene expression, butyrate promoted the transcription of thermogenic markers in 3T3-L1 adipocytes and SVF-derived primary adipocytes. Meanwhile, TMP269, a selective class IIa HDAC inhibitor that can effectively suppress the transcriptional activity of HDAC9 [32], showed comparable effects to butyrate. These data suggest that butyrate is capable of inhibiting HDAC9 and promoting adipocyte thermogenesis.

HDAC9 is a member of Class IIa HDACs, which possess a highly conserved C-terminal catalytic domain with weak deacetylase activity and an N-terminal regulatory domain facilitating the interaction with transcription factors [33]. HDAC9 stands out as a key modulator that can shuttle between the cytoplasm and nucleus to control gene expression. It is widely involved in cell proliferation, differentiation, inflammation, angiogenesis, glucose, and lipid metabolism [34]. Accumulating evidence has highlighted the detrimental roles of HDAC9 in atherosclerosis [35], liver fibrosis [36], autoimmune disease [37], cancer [38], and Alzheimer’s disease [39] in both humans and mice. Moreover, HDAC9 has been shown to act as a negative regulator of adipogenesis [40], and genetic deletion of HDAC9 increases energy expenditure and adaptive thermogenesis, consequently protecting mice from high-fat diet-induced obesity, insulin resistance, and liver steatosis [41]. In contrast, transgenic overexpression of HDAC9 induces adipocyte hypertrophy and insulin resistance in aging mice [42].

In this study, we show that butyrate downregulates HDAC9 expression in sWAT and adipocytes. In line with our research, previous studies also reported that butyrate can inhibit HDAC9 in blood monocytes [43] and erythroleukemia cells [44], while other SCFAs (including acetate and propionate) have been shown to reduce HDAC9 in T cells [45], monocytes [43], and keratinocytes [46]. Given the inhibitory effects of butyrate on HDAC9 and these important roles of HDAC9 in physiological processes and disease development, HDAC9 inhibition is emerging as a promising strategy to treat chronic diseases such as stroke, atherosclerotic cardiovascular disease [34], kidney fibrosis [47], depression [48], and obesity. Of note, to avoid side effects, more tissue-specific pharmacological inhibitors of HDAC9 are urgently needed.

In conclusion, this study verifies the benefits of butyrate on obesity prevention, which is explained by decreased food intake and increased adaptive thermogenesis in BAT and WAT. More importantly, we reveal HDAC9 as a new target of butyrate in promoting the browning of white adipocytes, providing HDAC9 inhibition as a promising strategy to treat obesity and related complications. Nevertheless, this study has some limitations. First, the specific role of HDAC9 in the anti-obesity effect of butyrate in vivo has not been shown. This could be further assessed using an adipose-specific HDAC9 knockout mouse model in our future study. Besides, the exact molecular mechanism of how HDAC9 inhibition promotes the expression of thermogenic genes is not clarified. We speculate that butyrate downregulates HDAC9, resulting in the histone acetylation and transcriptional modulation of key thermogenic markers such as *Pgc1a* and *Ucp1*. As a result, WAT browning is initiated to burn extra energy via UCP1-mediated heat production. This speculation is expected to be explored in our later work. Furthermore, given that various HDAC members are implicated in adaptive thermogenesis and can function synergistically, the redundancy effects of HDACs should be considered when elucidating the underlying molecular mechanisms.

## Data Availability

Data are available by contacting the corresponding author.

## Supplementary Materials

The following supporting information can be downloaded at: https://www.mdpi.com/article/10.3390/biomedicines13020260/s1, Table S1: primers for qPCR. Figure S1: Butyrate supplementation increases energy expenditure. Figure S2: Butyrate supplementation affects sWAT function. Figure S3: Butyrate supplementation markedly remodels the global transcriptome landscape of iBAT. Figure S4: Butyrate and the HDAC9 inhibitor TMP269 has little effects on mature adipocytes.
