# Supplementary material for: Butyrate Prevents Obesity Accompanied by HDAC9-Mediated Browning of White Adipose Tissue
Source: Biomedicines. 2025 Jan 21;13(2):260. doi: 10.3390/biomedicines13020260 (PMC11852213; doi:10.3390/biomedicines13020260)
Supplement: Supplementary file 1 [file biomedicines-13-00260-s001.zip › biomedicines-3344816-supplementary-revision.pdf]

Table S1 Primers for q-PCR

Primer name	Forward primer sequence (5'-3')	Reverse primer sequence (5'-3')
Ucp1	TCAGGATTGGCCTCTACGAC	TGCATTCTGACCTTCACGAC
Pgc1a	TGCTAGCGGTTCTCACAGAG	AGTGCTAAGACCGCTGCATT
Dio2	CCTGCCAGTCTTTTTCTCCA	ACGTGCACCACACTGGAAT
Cidea	CTCGGCTGTCTCAATGTCAA	CCGCATAGACCAGGAACTGT
Prdm16	ACTTTGGATGGGAGCAGATG	CTCCAGGCTCGATGTCCTTA
Hadc1	CTTACGAAACAGCGGTG	CTTCTCCAGGTACTCGTTA
Hadc2	TGCTGAAGAAATGACTAAATACC	CAAAGAGTCCATCAAACAC
Hadc3	AATGTGCCCTTACGAGATGG	GTAGCCACCACCTCCCAGTA
Hadc4	GCCTTCAGAACGGTGGTTAT	TACCCAAAACATTTGGCAGA
Hadc5	AGTGAGAGCACCCAGGAAGA	GTACACCTGGAGGGGCTGTA
Hadc6	TCCACCGGCCAAGATTCTTC	CAGCACACTTCTTTCCACCAC
Hadc7	TGAAGAATGGCTTTGCTGTG	CACTGGGGTCCTGGTAGAAA
Hadc8	AGGTGATGAGGACCATCCAG	ACCCTCCAGACCAGTTGATG
Hadc9	AGGATGATGATGCCTGTGGTGGAT	GAGTTGTGCTTGATGCTGCCTTGT
Hadc10	CAGAGGAAGAGTTGGGCTTG	GGTGTCCGGGTGAAAGTAGA
Pparg2	TCTGGGAGATTCTCCTGTTGA	GGTGGGCCAGAATGGCATCT
Cebp/b	TGGACAAGCTGAGCGACGAG	TGTGCTGCGTCTCCAGGTTG
Cebp/a	GGACAAGAACAGCAACGAGTA	GCAGTTGCCATGGCCTTGA
Cycophllln A	TATCTGCACTGCCAAGACTGAGTG	CTTCTTGCTGGTCTTGCCATTCC
aP2	ACACCGAGATTTCCTTCAAACTG	CCATCTAGGGTTATGATGCTCTTCA
Adiponectin	GCACTGGCAAGTTCTACTGCAA	GTAGGTGAAGAGAACGGCCTTGT
CD36	GCAAAGAACAGCAGCAAAATC	CAGTGAAGGCTCAAAGATGG
ATGL	ACAGTGTCCCCATTCTCAGG	TTGGTTCAGTAGGCCATTCC
HSL	AGACACCAGCCAACGGATAC	ATCACCCTCGAAGAAGAGCA
Glut4	GTGACTGGAACACTGGTCCTA	CCAGCCACGTTGCATTGTAG
Fasn	GGAGGTTGCTTGGAAGAG	CTGGATGTGATCGAATGCT

Figure S1

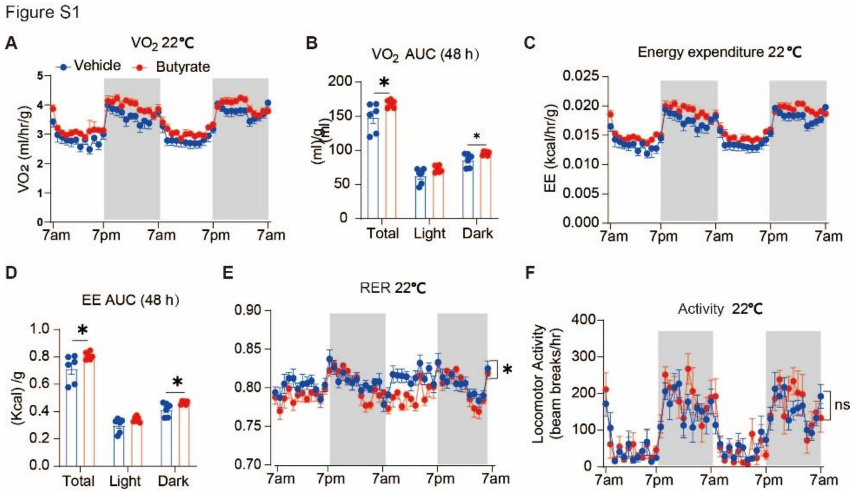

Figure S1. Butyrate supplementation increases energy expenditure. After 7 weeks of HFD intervention with or without butyrate, mice were housed in a Promethion metabolic measurement system at 22°C. After 3 days acclimatization, the oxygen consumption (A-B), energy expenditure (C-D), respiratory exchange ratio (RER; E) and physical activity (F) were monitored and calculated. Data are shown as means ± SEM (n=6) during a 48-hour cycle. Unpaired two-tailed Student's t test was used in B and D. Two-way ANOVA was used in E and F. *$P<0.05$.

Figure S2

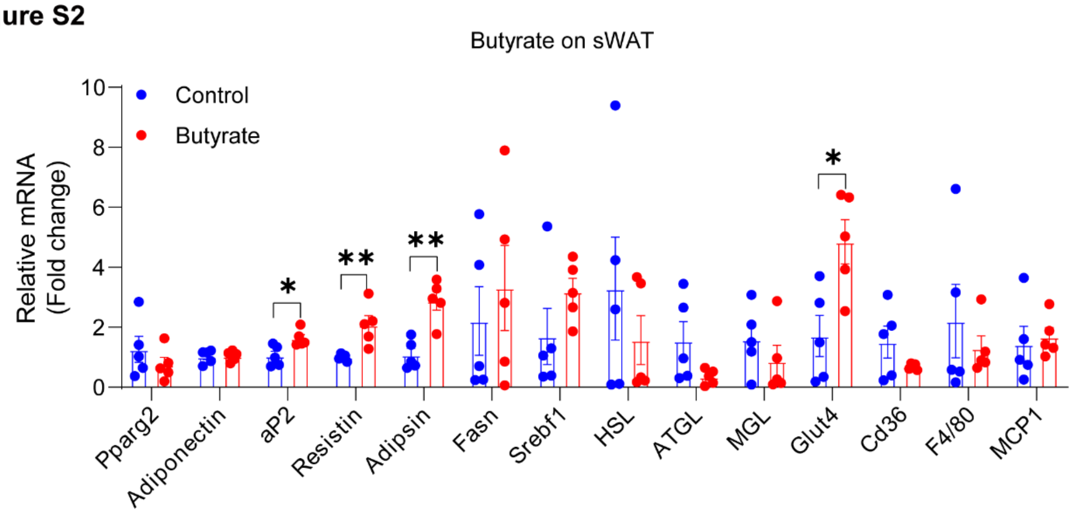

Figure S2. Butyrate supplementation affects sWAT function. After 9 weeks intervention, mRNA expression of adipogenesis, adipokine, lipogenesis, lipolysis, glucose uptake, fatty acid transportation and inflammation were analyzed using q-PCR in sWAT. Data were shown as means ± SEM (n=5). Unpaired two-tailed Student's t test was used. *P<0.05, **P<0.01.

Figure S3

Figure S3

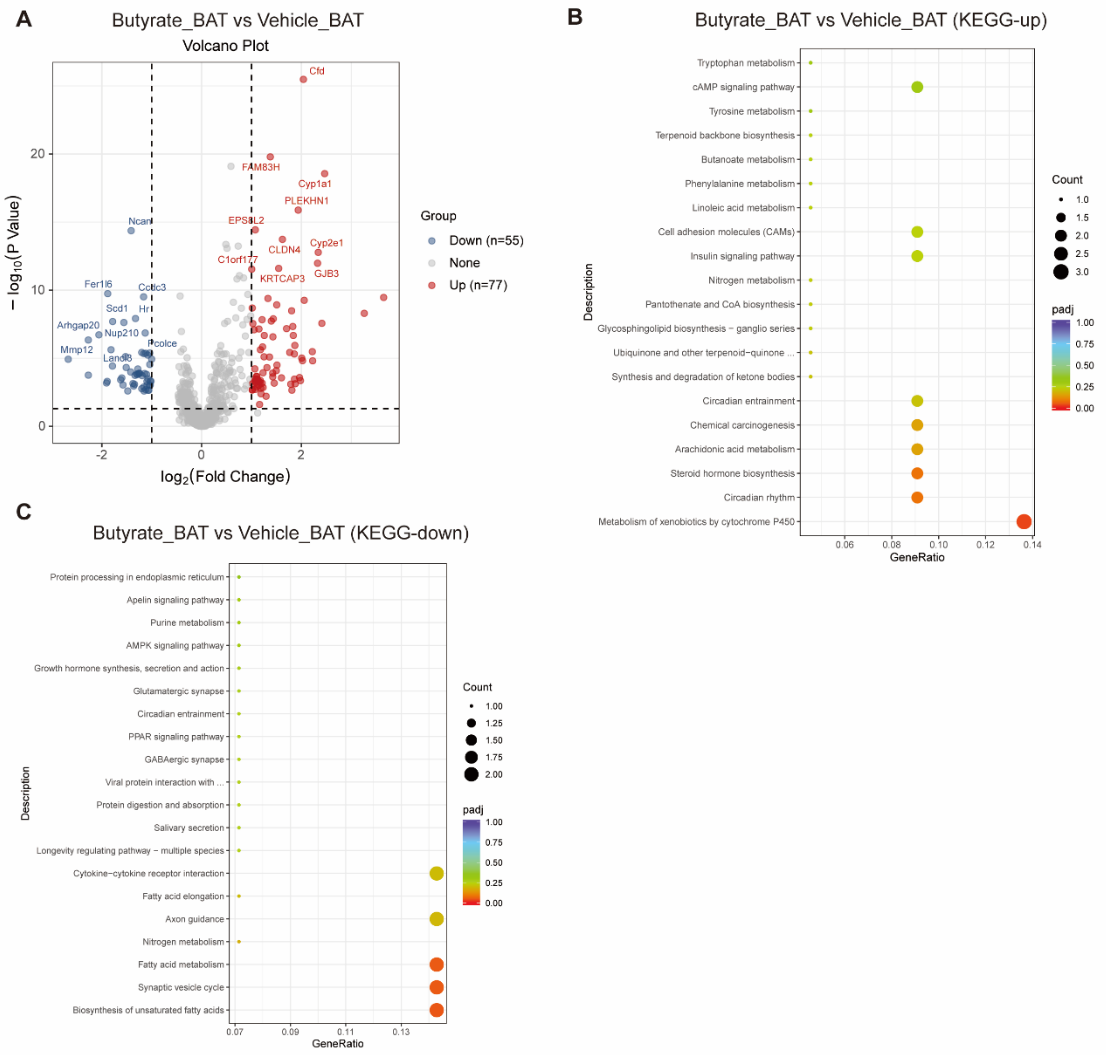

Figure S3. Butyrate supplementation markedly remodels the global transcriptome landscape of iBAT. Mice were fed a high-fat diet and treated with or without butyrate for 9 weeks. Heat map depicting the profile of differential gene expression in iBAT (A). Enriched KEGG terms of iBAT that were significantly higher expressed in butyrate group (B). Enriched KEGG terms of iBAT that were significantly lower expressed in butyrate group (C).

Figure S4

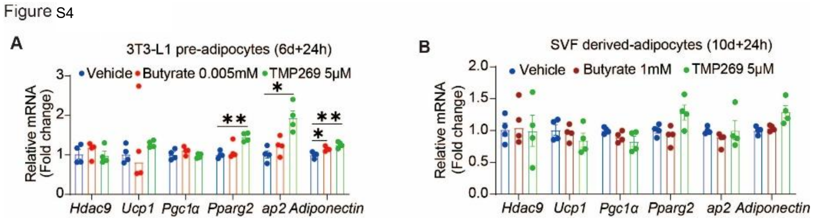

Figure S4. Butyrate and the HDAC9 inhibitor TMP269 had little effects on mature adipocytes. 3T3-L1 pre-adipocytes were differentiated for 6 days and then treated with PBS, 5 μM butyrate or 5 μM HDAC9 inhibitor TMP269 for 24 h, mRNA levels were analyzed by q-PCR (A). Stromal vascular fraction (SVF) of mouse sWAT was isolated and cultured to induce adipocytes for 10 days and then treated with PBS, 1mM butyrate or 5 μM TMP269 for 24 h, mRNA levels were analyzed by q-PCR (B). Data were shown as mean ± SEM (n=4). One way ANOVA was used. *P<0.05, **P<0.01.
